# Comparison of the Hemagglutination Inhibition Test and IgG ELISA in Categorizing Primary and Secondary Dengue Infections Based on the Plaque Reduction Neutralization Test

**DOI:** 10.1155/2016/5253842

**Published:** 2016-06-30

**Authors:** Nurhayati Lukman, Gustiani Salim, Herman Kosasih, Nugroho Harry Susanto, Ida Parwati, Silvita Fitri, Bachti Alisjahbana, Susana Widjaja, Maya Williams

**Affiliations:** ^1^Indonesia Research Partnership on Infectious Diseases (INA-RESPOND), Jalan Percetakan Negara No. 29, Jakarta 10560, Indonesia; ^2^U.S. Naval Medical Research Unit No. 2, Jakarta 10560, Indonesia; ^3^Health Research Unit, Universitas Padjadjaran, Jalan Eijkman No. 38, Bandung 40161, Indonesia; ^4^Clinical Pathology Department, Hasan Sadikin Hospital, Faculty of Medicine, Universitas Padjadjaran, Jalan Pasteur No. 38, Bandung 40161, Indonesia

## Abstract

Secondary dengue infection by heterotypic serotypes is associated with severe manifestations of disease, that is, dengue hemorrhagic fever (DHF) and dengue shock syndrome (DSS). The World Health Organization (WHO) has recommended criteria based on the hemagglutination inhibition (HI) test to distinguish between primary and secondary dengue infections. Since the HI test has practical limitations and disadvantages, we evaluated the accuracy of WHO HI criteria and compared it with criteria based on an IgG enzyme-linked immunosorbent assay (ELISA) using a plaque reduction neutralization test (PRNT) as the gold standard. Both WHO HI criteria and IgG ELISA criteria performed strongly (16/16) in determining primary infection. However, to determine secondary infection, the IgG ELISA criteria performed better (72/73) compared to the WHO HI criteria (23/73).

## 1. Introduction

Dengue virus is a global concern with an increasing incidence, especially in endemic areas like Southeast Asia, South America, and the Pacific. Recent analysis, based on the geographical distribution of the disease, estimates 250–500 million infections annually, which is three times higher than the estimation from the World Health Organization (WHO) [1, 2].

Dengue virus (DENV) belongs to the family Flaviviridae, genus* Flavivirus*, which consists of four antigenically distinct serotypes (DENV-1, DENV-2, DENV-3, and DENV-4). Dengue virus infection can be confirmed by virus culture, by reverse-transcription polymerase chain reaction (RT-PCR), serologically by neutralization test (gold standard), by IgM enzyme-linked immunosorbent assay (ELISA) antibodies, or by 4-fold increase of hemagglutination inhibition (HI) antibody titers between acute and convalescent specimens. HI test is more commonly used than PRNT as HI test does not need virus culture facilities and has a simpler technique [3].

Infection with any of the four serotypes can be asymptomatic or results in a wide range of clinical manifestations between dengue fever (DF), mild to severe dengue hemorrhagic fever (DHF), and dengue shock syndrome (DSS) which is often fatal. Besides viral virulence and host background (immune, genetic, and nutritional), severe dengue infection is often associated with secondary infections [4]. Secondary infections, which are common in endemic areas such as Indonesia [5–8], comprised approximately 80–90% of the DHF/DSS cases. Therefore, it is important to distinguish between primary and secondary infections. The 2009 WHO guidelines on dengue suggested criteria based on HI titers in convalescent sera to distinguish between primary and secondary infection. While the accuracy of the WHO HI criteria in distinguishing between primary and secondary infections has been questioned [9–14], these criteria are still used in studies which analyze the relationship between primary or secondary infections, disease severity, and studies conducted to develop serological methods [9, 10, 15–17]. Several methods based on ELISA, which are easier and faster to perform, have been proposed as alternative methods to distinguish between primary and secondary infections, for example, by measuring IgG antibodies; the avidity titer of IgG; calculating the ratio of IgM/IgG; or the absence of IgG in acute specimens [11, 12, 14, 18, 19]. However, none of these studies utilized another serological assay as the gold standard. In this study, we compared the utility of the WHO HI criteria and IgG ELISA in differentiating primary and secondary infections using acute and convalescent specimens that had been tested by a plaque reduction neutralization test (PRNT). In addition, we also propose modified HI criteria to distinguish between primary and secondary infections.

## 2. Materials and Methods

### 2.1. Sample Collection

This study used 178 acute and convalescent sera from 89 confirmed dengue cases. These sera were collected following written informed consent as a part of two dengue studies in Bandung. These studies were approved by the Institutional Review Board of the United Stated Naval Medical Research Unit (US-NAMRU)#2 (N2.2006.0001 and N2.2004.0010) in compliance with all US Federal Regulations governing the protection of human subjects and at the National Institute of Health Research and Development, Ministry of Health, Indonesia (KS.02.01.2.1.2776 and KS.02.01.2.1.3461). Study procedures were done in accordance with the Helsinki Declaration of 1975, as revised in 2013.

Acute sera were taken when study participants came to health centers or hospitals, while convalescent sera were obtained at least seven days later or when the participants were discharged from the hospitals.

### 2.2. Dengue Laboratory Assays

A dengue infection was confirmed when (1) DENV was isolated from tissue culture or DENV RNA was detected by the RT-PCR or (2) when the acute sample was positive for IgM and paired sera demonstrated increasing IgG titers and/or a fourfold rise in HI titers. Primary or secondary infection was confirmed by PRNT, IgG ELISA, and HI tests.

#### 2.2.1. Dengue IgM ELISA Test

Dengue IgM tests were conducted using commercial ELISA kits (Dengue IgM ELISA, Focus Technologies, Cypress, CA, USA). The tests were performed following manufacture's procedures as previously described [20].

#### 2.2.2. Dengue IgG ELISA Test

IgG ELISA was performed using a commercial dengue IgG ELISA kit (Dengue IgG ELISA, Focus Technologies, Cypress, CA, USA), following the manufacturer's instructions. Index value was calculated by dividing sample optical density (OD) with mean of calibrator OD. Specimens with an IgG index value <1 were confirmed as negative and those with IgG index value >1 were confirmed as positive. Dengue case with negative IgG results from the acute specimen was classified as a primary infection.

#### 2.2.3. Dengue HI Test

HI test was performed according to the procedures [21]. A preliminary experiment comparing the performance of all four dengue antigens in the HI test did not show any difference in their ability determining recent infection or in differentiating primary and secondary infections (data not shown). Hence, only dengue-3 antigen was used for HI test throughout the rest of this study. Specimens with detectable HI antibodies at 1 : 10 or higher dilutions were confirmed as positive. If no antibody was detected at the lowest dilution (1 : 10), then the specimen was confirmed as negative.

WHO recommended using HI titers of convalescent sera as the criteria to distinguish between primary and secondary infection. Dengue infection with convalescent HI antibody titer higher than 1280 was classified as a secondary infection [1].

#### 2.2.4. PRNT

Plaque reduction neutralization test (PRNT) was performed using the following reference strains: DENV-1 (16001), DENV-2 (16682), DENV-3 (16562), and DENV-4 (1036). In brief, 500 plaque forming units per milliliter of dengue virus were incubated with serial 4-fold diluted serum from 1 : 10 to 1 : 10, 240. A suspension of the baby hamster kidney (BHK) cells was used to detect the virus reduction following previously described methods [22]. PRNT_50_ titer was calculated using a log probit regression analysis by SPSS (SPSS Inc., Chicago, IL). Negative result for neutralizing antibodies was confirmed when PRNT_50_ values were lower than 10 against all dengue serotypes. A positive result for neutralizing antibodies was confirmed when PRNT_50_ titers were 10 or greater for at least one dengue serotype. Cases with seroconversion of neutralizing antibodies were classified as a primary infection; and cases with detectable neutralizing antibodies in acute specimen were confirmed as a secondary infection.

### 2.3. Statistical Analysis

Statistical analysis was performed using STATA version 9 (STATA Corporation, TX). Comparison between the two assays was analyzed using McNemar's test.

## 3. Results

Subject demographics and specimen characteristics categorized by primary and secondary infection according to PRNT results are presented in Table 1. While subjects with a primary infection were slightly younger than those subjects with a secondary infection, there was no significant difference in terms of age, gender distribution, acute sample collection day, convalescent sample collection day, or the interval between acute and convalescent sample collection.

### 3.1. HI and IgG ELISA Performances Compared to PRNT

First, we sought to examine the sensitivity and specificity of IgG ELISA and HI test in comparison to PRNT. Based on PRNT results, 162 of 178 specimens were positive for dengue neutralizing antibodies and 16 specimens (all acute) were negative. Among the 162 positive samples, IgG ELISA results were positive for 161 (99.4%) samples and HI test results were positive for 153 (94.4%) samples. Among the 16 specimens confirmed negative by PRNT, IgG ELISA results were also negative for these samples, whereas HI test results were negative in only 10 (62.5%) samples. Thus, while the sensitivities between ELISA and HI test were similar compared to PRNT, the IgG ELISA was significantly more specific than the HI test (*p* < 0.01).

### 3.2. Distinguishing between Primary and Secondary Infections

Next, we aimed to determine the performance of IgG ELISA and WHO HI criteria in distinguishing between primary and secondary infection using titer from convalescent samples with PRNT results as the gold standard. According to PRNT results, from 89 dengue cases included in this study, 16 cases were categorized as a primary infection and 73 cases were categorized as a secondary infection. Results from both HI test using convalescent samples and IgG ELISA using acute samples concurred in all of the 16 primary cases. IgG ELISA also identified 72 of 73 (98.6%) secondary cases, whereas HI test was only able to detect 23 of the 73 (31.5%) secondary cases. The difference between the two results was significant (*p* < 0.01).

In Table 2, our results show that 11 cases with convalescent HI titers ≤80 were all confirmed as a primary infection by PRNT. In 32 cases with convalescent HI titers between 160 and 640, which are considered as a primary infection by WHO criteria, PRNT results only confirmed five (15.6%) cases. All 46 cases with convalescent HI titers ≥1280 were also confirmed as a secondary infection by PRNT.

To determine if we could better categorize the cases with convalescent HI titers between 160 and 640, we considered HI results from the acute samples of these cases. Cases with convalescent HI titers between 160 and 640 and negative HI titer in acute samples were considered as a primary infection. Cases with convalescent HI titers between 160 and 640 and positive HI titers in acute samples were considered as a secondary infection (Table 3, modified HI-1).

Applying this modified classification, 2 of the 5 cases in confirmed primary infection by PRNT did not have HI antibodies in the acute samples, whereas 22 of 27 cases in confirmed secondary infection by PRNT had HI antibodies in the acute specimens. Hence, these 24 cases were classified in concordance with PRNT results. Using this criteria, 13/16 (81.3%) primary cases and 68/73 (93.2%) secondary cases were classified in concordance with PRNT results. We also calculated based on acute sample HI results for classification by defining cases with no HI antibodies in acute samples as primary infections and cases with HI antibodies in acute samples as secondary infection (Table 3, modified HI-2). Using this criteria, 10/16 (62.5%) primary cases and 64/73 (87.7%) secondary cases were classified in concordance with PRNT results.

Table 3 summarizes the percentage of cases classified in concordance with PRNT results as a primary or a secondary infection using all of the criteria examined in this study. Cases classified according to acute sample IgG ELISA results had the highest concordance with PRNT classification, while criteria that took into account both convalescent and acute HI results had the next best concordance with PRNT classification. The performance in identifying primary infection is statistically different between modified HI-2 and IgG ELISA and modified HI-2 and WHO HI criteria. The performance in identifying secondary infection is statistically different between WHO HI and all other criteria. It is also significantly different between IgG ELISA and modified HI-2.

## 4. Discussion

Serology assays are the most applicable and affordable confirmatory test for dengue infections in third world countries where dengue is commonly endemic. In addition, WHO has recommended IgM Antibody Capture (MAC) ELISA, HI test, and PRNT to determine between primary and secondary infection [21]. Compared to MAC ELISA and PRNT, HI test has been the most widely used serology assay for a long time. However, similar to previous reports, we showed that HI test underestimated secondary infection when the WHO recommended cutoff was applied [13, 14]. The low performance of the HI test in secondary infection has also been reported previously by other groups. The HI antibodies do not raise to reach the titer of 1 : 1280, as required by the WHO HI criteria [14]. This might be associated with the degradation of a fraction of HI antibodies which is below the test detection threshold [13]. By applying modified cutoff criteria with both convalescent and acute sample HI results, the accuracy in determining secondary infection increased from 31.5% to 93.2%, while the accuracy in determining primary infection decreased from 100% to 81.3%.

Nevertheless, compared to the HI test, the commercially available ELISA kit is simpler and faster than the HI test. Thus ELISA kit is easier to obtain than HI antigens in some countries such as Indonesia. Although several types of ELISA evaluation (avidity, ratio IgM/IgG) have been compared with HI and have been determined to be more reliable than HI tests, these studies did not use a third assay as a reference [12, 14, 18, 19]. By using PRNT as the gold standard, our study has confirmed results from previous studies that ELISA is superior to HI in determining primary and secondary infections.

Our study was similar to de Souza et al. in regard to the IgG ELISA kit used (Focus Diagnostics) and the simple criteria for determining between primary and secondary infection—the absence or presence of IgG antibodies in the acute specimens [19]. As other IgG ELISA kits may have different sensitivities and specificities, these criteria should only be used after the sensitivity and specificity of these kits are assessed. Compared to other methods that used IgG ELISA and showed similar performance, such as IgM/IgG ratio and avidity index, our method is simpler and more cost-effective.

## 5. Conclusion

Considering the limitation of HI criteria in differentiating between primary and secondary infection, we recommend the use of the absence or presence of IgG antibodies in acute specimens as measured by IgG ELISA as the criteria. HI may still be used; however, the criteria for interpretation should be revised. We propose that HI convalescent titers ≤80 should be classified as primary infections, HI convalescent titers ≥1280 should be classified as secondary infections, and, for cases with HI convalescent titers between 160 and 640, the acute samples should be evaluated. Cases with acute sample that does not have any detectable HI antibodies should be classified as a primary infection, while cases with acute sample that have detectable HI antibodies should be classified as a secondary infection. This study shows that performance of acute IgG ELISA criteria and the proposed HI criteria is equally good.

## Figures and Tables

**Table 1:** Subject demographics and sample characteristics.

	Subjects		Samples		
	Age (years^*∗*^)	Male (%)/female (%)	Acute (days of onset^*∗*^)	Convalescent (days of onset^*∗*^)	A-C interval (days^*∗*^)
Primary (*n* = 16)	26 ± 9, (7–42)	13 (81)/3 (19)	2.5 ± 1.7, (1–7)	18.5 ± 5.9, (9–32)	15.9 ± 5.6, (7–30)
Secondary (*n* = 73)	36 ± 8, (9–53)	47(64)/26 (36)	2.1 ± 1.3, (1–7)	15.3 ± 4.4, (6–28)	13.2 ± 3.9, (4–26)

Total (*n* = 89)	34 ± 9, (7–53)	60 (67)/29 (33)	2.2 ± 1.4, (1–7)	15.9 ± 4.8, (6–32)	13.7 ± 4.4, (4–30)

^*∗*^Mean ± SD (range).

**Table 2:** Convalescent HI titers and case classification according to PRNT.

HI titers	Cases	PRNT			
		Primary infection		Secondary infection	
		Cases	%	Cases	%
≤80	11	11	100.0%	0	0.0%
160	4	1	25.0%	3	75.0%
320	14	3	21.4%	11	78.6%
640	14	1	7.1%	13	92.9%
1280	23	0	0.0%	23	100%
≥2560	23	0	0.0%	23	100%

**Table 3:** Percentage of cases classified in concordance with PRNT classification, using IgG ELISA, WHO HI criteria, or modified HI criteria.

	IgG ELISA	WHO HI criteria	Modified HI-1^a^	Modified HI-2^b^
Primary infection	16/16 (100)	16/16 (100)	13/16 (81.25)	10/16 (62.5)
Secondary infection	72/73 (98.6)	23/73 (31.5)	68/73 (93.2)	64/73 (87.7)

^a^Primary infection if convalescent HI antibody titers are ≤80 or if convalescent HI antibody titers are 160, 320, or 640 and HI antibody titers negative in acute specimen; ^b^primary infection if HI antibody titers are negative in acute specimen.
